# Optimized Feature Engineering and Machine Learning Approaches for Early Prediction of Renal Cell Carcinoma

**DOI:** 10.3390/cancers18203384

**Published:** 2026-10-19

**Authors:** Neha Chankhore, Deepali Vora, Paolo Barsocchi, Akash Kumar Bhoi

**Affiliations:** 1 Symbiosis Institute of Technology, Pune Campus, Symbiosis International (Deemed University), Pune 412115, India; chankhoreneha16@yahoo.com (N.C.); akashkrbhoi@gmail.com (A.K.B.); 2 Institute of Information Science and Technologies, National Research Council, 56124 Pisa, Italy

**Keywords:** renal cell carcinoma, machine learning, SHAP, clinical features

## Abstract

Renal cancer is a fatal disease and ranks among the 14 common cancers in the latest WHO ranking. Background/Objectives: Renal cell carcinoma (RCC) prediction not only diagnoses the disease at early stages but also prevents patients from suffering pain and eventual death. This study aimed to construct and validate a model that predicts renal cancer using machine learning (ML) classifiers. Methods: Feature engineering techniques were used to refine the dataset, such as interaction feature generation, which categorizes the features according to values such as BMI, eGFR, and radiographic size. Binary clinical risk flags identify clinical risks according to the feature values, and correlation pruning removes the common and correlated features among various features. Three public datasets were collected, and the performance of seven machine learning classifiers was evaluated using the proposed model. The evaluation parameters included accuracy, precision, recall/sensitivity, specificity, F1 score, and Matthews Correlation Coefficient (MCC). An Explainable AI method was used to visualize the importance of the model characteristics. Results: Among all the algorithms, the Multilevel Perceptron (MLP) algorithm fits best for the prediction of renal cancer with 98%, 96%, and 100% accuracy, and SHapley Additive exPlanations (SHAPs) are used to visualize the importance of all model features. Conclusions: The proposed model provides an efficient method and shorter computation time to predict renal cancer using clinical features.

## Simple Summary

Renal cancer ranks 14th amongst most fatal cancer diseases listed by WHO worldwide. Apparently, pre-diagnosis of renal cancer plays a role in accurate treatment and a plan of treatment to save the patient can be suggested. The study aims to access the patient information from three different datasets to analyze the prediction of disease using clinical information. The feature engineering techniques will filter the dataset to standardize the prediction process, whereas XAI will identify which features from all the datasets are essential in prediction. The Multilevel Perceptron proves best amongst the algorithms in the prediction of renal cancer with highest accuracy. SHapley Additive exPlanations (SHAPs) visualize the comparison of the features and its impact.

## 1. Introduction

Renal cancer, renal cell carcinoma (RCC), accounts for 2% to 3% of global cancer diagnoses and mortality. It contributes to 80–85% of all renal cancers [1]. Chronic cancers impact global health statistics and challenge healthcare systems. Long-term diseases such as cardiovascular disease, heart health, diabetes, kidney disease, and high blood pressure impact the health of a person and eventually result in chronic diseases. Aging and poor dietary habits have resulted in chronic renal cancer. Long-term smoking habits impact not only the lungs but also vital organs. Similarly, tobacco gradually affects the vital organs of the body. Therefore, the early detection of chronic diseases will play a pivotal role in the survival of patients, and timely medication and surgery are suggested as per disease progression. Chronic renal cancer is asymptomatic, and patients fail to identify the symptoms until it worsens. The severe symptoms experienced by patients in the final stages of cancer were high fever, blood in urine, and fatigue due to dehydration. A delay in identifying symptoms may lead to eventual death or a lower survival rate. Therefore, there is an urgent need for a model that can predict renal cancer in the early stages.

This research paper aims to identify multiple datasets which have clinical data and apply a hybrid machine learning algorithm for renal cell carcinoma. The literature study identifies various machine learning algorithms used in prediction of renal cell carcinoma. The study suggests a hybrid feature extraction and selection model that can be used for the prediction of renal cell carcinoma using feature engineering applied on various machine learning algorithms.

The research paper comprises clinical input data for model validation. Data preprocessing techniques use various methodologies to clean the data for further processing. Feature engineering methods were used to refine the extracted features for prediction. Machine learning classifiers were used for the final prediction of the model. This study proposes a model for renal cancer prediction using the following steps:

- The various datasets were preprocessed for renal cancer prediction.
- Feature engineering was used for feature scaling, categorical encoding, finding interactive features, and missing value engineering.
- To implement machine learning classifiers for the prediction of renal cancer.
- To visualize the important features identified using Explainable AI (XAI).

The remainder of this paper is organized as follows: The second section presents a literature review of the methodologies used. The third section describes the dataset. The fourth section includes a detailed description of the methodologies used in the research. The fifth section presents the experimental setup, including the architecture of the research and the methodologies used in the research. The sixth section presents the results and discussion, summarizing the results obtained and discussing the methodology and results. The seventh section concludes with the overall work and future scope of the study. The eighth section is a list of references.

## 2. Literature Survey

Feature engineering incorporates the refinement of features using feature selection and processing of the dataset for filtration and generation of data. Machine learning methodologies in the healthcare domain have been proven to deliver the highest predictable results in a cost-effective manner. The use of Explainable AI (XAI) for the prediction of renal cancer proved beneficial for the explanation of the use of predictive models and feature importance. The use of AI in healthcare helps enhance the prediction results and, eventually, the overall model performance. R. Sawhney et al. [2] represents a comparative study of machine learning algorithms, including the MLP classifier, which predicts the result with 99% accuracy. The model predicts 25 clinical features, and an Artificial Neural Network (ANN) proves to be the best fit with 100% accuracy. Feature selection techniques, such as Principal Component Analysis (PCA) and correlation analysis, are used. Md J. Raihan et al. [3] proposed an XGBoost classifier for chronic renal cancer prediction. The predicted model provides an accuracy of 99.16%, and with the use of the Biogeography-Based Optimization (BBO) algorithm, the accuracy increases to 98.33%. The impactful features used were represented using the SHAP analysis.

I. Ibrahim et al. [4] presents a multiprocessing interface of genetic algorithms for optimizing disease prediction. The MLP was processed by the multiprocessing genetic algorithm (MIGA) to achieve an accuracy of 100% for chronic renal cancer. The modified perturbed MLP model proposed by Wang et al. [5] outperformed traditional deep learning and Random Forest algorithms. The PMLP predicted the best accuracy of AUC 0.998 for bone, brain, liver, and lung metastases. For a comparative analysis of clinical data and images, Jie Xu et al. [6] compared the execution of machine learning algorithms with the highest accuracy of MLP with an AUC of 0.705 using clinical data. M.L. Jacquline et al. [7] used the hybrid model Enhanced Butterfly Optimization and Improved Artificial Neural Network (EBO-IANN) algorithm for the prediction of chronic renal cancer, and it gave the accuracy in the range of 95.9–98.9%.

Jamim et al. [8] compared multiple chronic diseases using two datasets from Abu Dhabi Hospital and India and showed prediction results of 96% and 100%, respectively, for chronic renal cancer. Sumon et al. [9] compared the MLP classifier with all machine learning classifiers for the classification of heart disease and used SHAP to highlight the features that contributed to improving the performance of the model. Wen H. et al. [10] explains the evaluation metrics and use of SHAP for key performance indicators.

Table 1 shows a review of the papers used to analyze the dataset for prediction, along with the feature selection and extraction methods used for refining the data and the classification method used for the prediction of the filtered dataset and its performance evaluation at the end. The Inferences section will infer the findings or gaps identified through the study. As per the gaps identified in the below table, the feature engineering technique is applied on all the datasets and results will be compared.

## 3. Dataset

The dataset used to generate the clinical data prediction model was obtained from various publicly available data sources. The Cancer Genome Atlas (TCGA) [16] dataset is available for cancer researchers worldwide. The Tawam Hospital in Al-Ain City (Abu Dhabi) [13] provides data from hospitals for cancer research, whereas the UCI India [12] dataset also has data available with patient details. The chronic kidney disease (CKD) dataset provides detailed clinical information regarding the kidney area rather than focusing on the complete renal area. The RCC on the other hand focuses on the renal area which contains the kidney, urinary bladder, ureters, urethra, renal pelvis, etc. The CKD condition is a main cause of RCC which identifies the kidney damage condition. Considering RCC in a broader perspective helps in identifying the severity of the kidney disease in prediction. The following dataset focuses mainly on the renal area which includes parameters like ‘eGFR’ which evaluate the Estimated Glomerular Filtration Rate. The following dataset was selected, which was available online.

### 3.1. The Cancer Genome Atlas (TCGA)

The TCGA Program offers researchers access to all cancer datasets to facilitate cancer research. The dataset has 60 features and 210 patient data, of which 37 features were finalized and 23 features were dropped as per the feature engineering. The accepted features included sex, disease history such as cardiovascular disease, renal cancer, smoking history, and vital status.

### 3.2. Tawam Hospital in Al-Ain City (Abu Dhabi)

The Tawam Hospital dataset provides access to clinical data for researchers in renal cancer prediction. The medical records of 491 patients from January 2008 to December 2008, including 241 women and 250 men, were reviewed. The dataset has 22 features of 491 patients, of which 21 features are considered, and one feature is dropped after feature engineering. The accepted features include sex, disease history such as diabetes, cardiovascular disease, obesity, and eGFR.

### 3.3. India

The UCI India dataset was collected from a hospital for chronic renal cancer prediction as CKD and NotCKD. The dataset consisted of 400 medical records collected over two months. The dataset has 26 features of 400 patients, of which 23 features were selected, and 3 features were dropped after feature engineering. The accepted features include age, sex, hemoglobin level, high-risk factors, and cancer status. The Table 2 below shows a comparison of the dataset with the distribution of dataset information.

## 4. Methodology

### 4.1. Architecture Diagram

**Figure 1:**
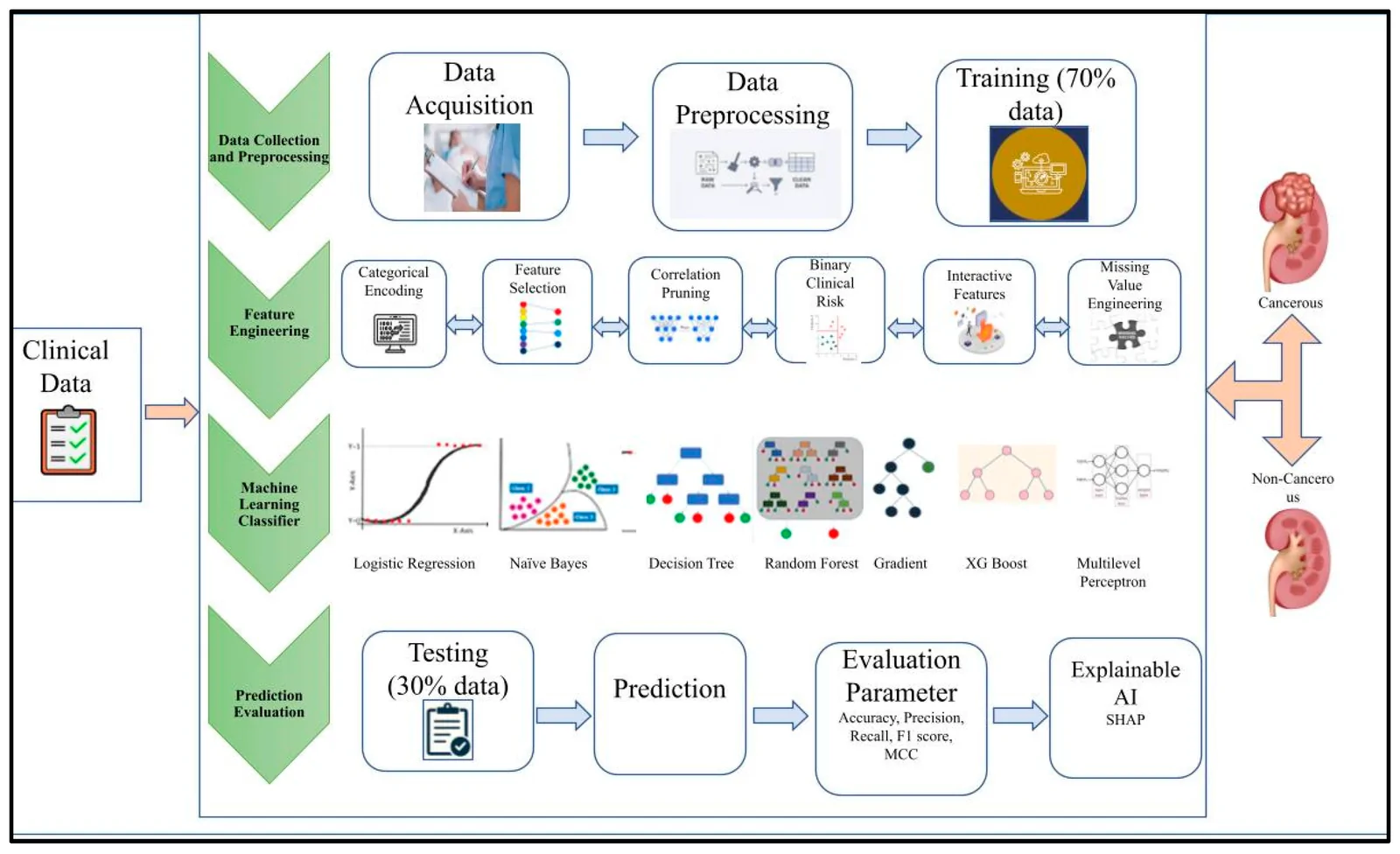
The detailed workflow of renal cancer prediction.

**Figure 2:**
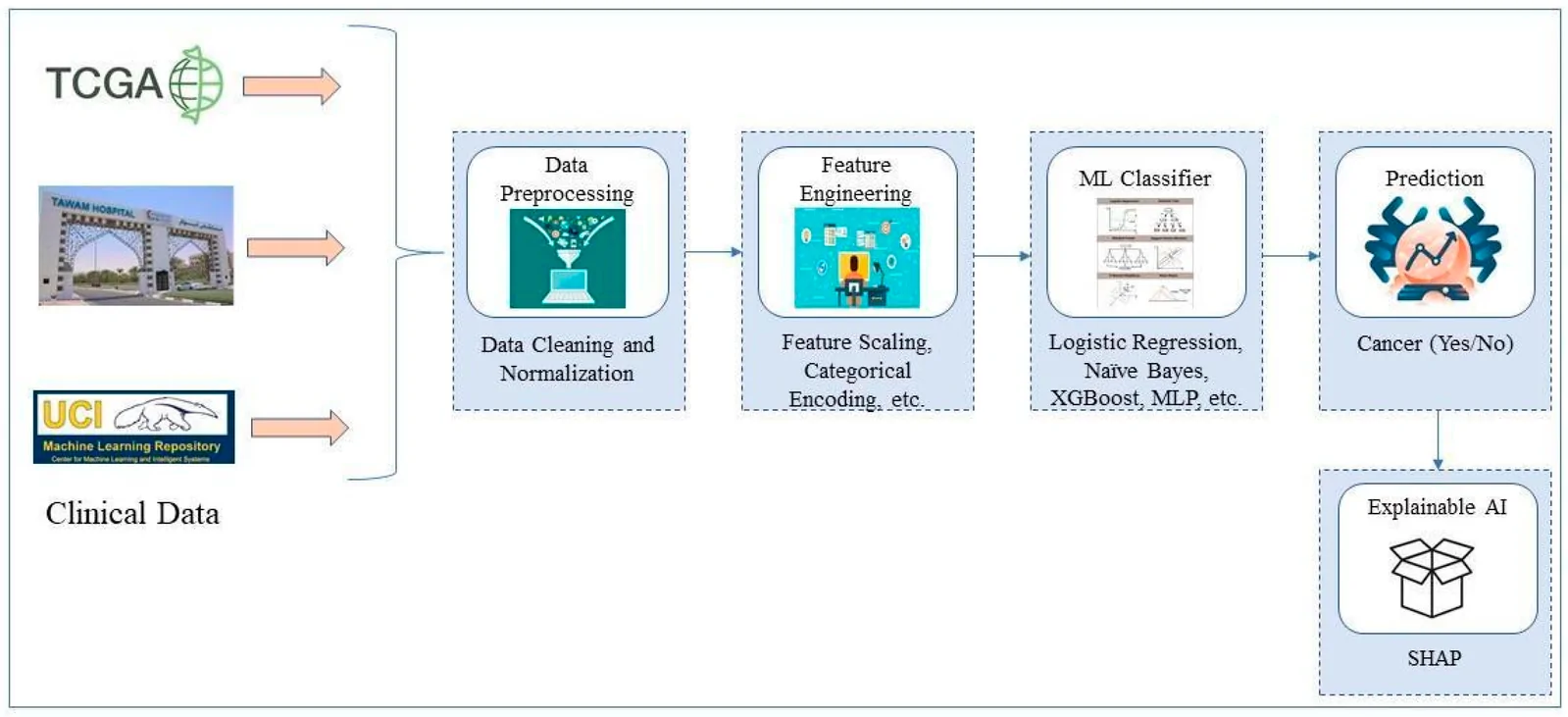
Renal cancer prediction workflow.

The architecture diagram in Figure 1 operates in three phases. The first phase involved data collection from public sources. The data acquisition technique was used to collect data from public sources, such as TCGA and Kaggle. Data Collection and Preprocessing involves the Data preprocessing cleans data by handling missing values, handling null values, and standard scaling. The second phase is feature engineering, where the preprocessed data are used to fine-tune further classification. Feature Engineering phase involves the techniques such as feature scaling and normalization will normalize the value in the standard format of the dataset, categorical encoding will encode a categorical value to numerical or vice versa, binary clinical risk flag will generate a binary risk in the form of 0 or 1 where 1 is the risk and 0 is the non-risk feature, interaction feature generation is used to generate the interactive feature from the existing vital features to generate interactive data for boosting the prediction results, feature selection technique will select the features which will be used for prediction of the data, correlation pruning will eliminate the correlated columns with similar values in multiple patients, etc. Whereas the training on 70% data is done after preprocessing to prepare a model and testing on 30% data is done once the feature engineering techniques and machine learning models are finalized to test the efficiency of models and compare the results. The third and final phase is the prediction phase with Machine Learning Classifier, where machine learning classifiers check the dataset and evaluate it according to the evaluation matrix. The selected features were trained on 70% of the data and tested on 30% of the data. Prediction Evaluation phase predicts the model percentage accuracy of the dataset. Finally, based on the comparison of all the model’s predictions, the cancer prediction was classified as cancer yes or no. Figure 2 shows the detailed workflow of renal cancer prediction.

The proposed model processes tabular clinical data. The renal cancer prediction workflow is shown in Figure 2.

Clinical data were used as input for the model. The model uses tabular clinical features, which refine the dataset and are used for further processing and eventually the prediction of the model. The clinical dataset consisted of patient details from the start of treatment to the identification of the disease. The proposed process refines the features and finalizes them based on the target prediction. Explainable AI visualizes the features of its importance in prediction.

### 4.2. Preprocessing of Clinical Data

In the field of predictive analysis, the dataset used is quality data, and the significance of the features should be preserved. The diverse dataset may lead to variations in features and their values. The preprocessing technique transforms the data into a clean and structured format. These datasets may have missing values, categorical or non-categorical values, outliers, or irrelevant features that need to be identified. Designing a predictive model for the medical domain requires precise and high-quality data to ensure reliability and increase predictability.

Data preprocessing is used to preprocess the dataset according to the application requirements. Key features are extracted for the prediction of the disease. Refining the dataset includes handling missing values, handling outliers, and removing duplicate values with relevant values. These measures of preprocessing data should align with patient data and ethical considerations before processing.

Handling missing values in data preprocessing deals with the handling of empty rows, along with values such as NaN or Null. The empty rows were replaced with mean, median, or mode values with data type available for that feature. Handling outliers in data preprocessing will replace the numerical value with the mean to maintain data integrity, whereas the categorical value will be replaced with the mode. For example, before applying the feature engineering on the dataset, data preprocessing is done where outliers are handled by applying the mean to random values for features like ‘age’ which have random values as per the dataset. Whereas some of the categorical values are replaced with the mode in their missing fields for features like ‘pc’ and ‘pcc’ which have categorical values as ‘normal’ and ‘abnormal’. The use of preprocessing techniques ensures the accuracy of the prediction and relevance to finding the dataset.

Clinical data preprocessing includes handling missing values using imputation. The median values were imputed to mitigate the skewness effect, whereas the mode imputed categorical values. The ‘rbc’ column was replaced with the median of the other column values. In the proposed model, the median of the values is replaced using following equation:

*X_missing_* = *median*(*X*) (1)

Whereas when dropping the column, the following equation will be used to drop the null value columns, if any:

*Drop column if* = (*Missing Values*)/*Total* > 0.7 (2)

Here, 0.7 represents 70% of the data in the column. The standard scaling of features is used to scale the feature values using the mean and original value of features as follows:

(3) $$Standard\;Value=\frac{Original\;Value\;-\;Mean}{Standard\;Deviation}$$

The high-risk parameters where data is replaced with mean or median or mode were very few like ‘*pc*’, ‘*pcc*’, ‘*rbc*’, etc., which hardly cause any impact on the prediction of disease and eventually data leakage will have less impact. To avoid the data leaks, variables like heart failure rate, appetite, total duration of disease impact on person, etc., were excluded. The final predictor variables used included ‘*Age*’, ‘*Sex*/*Gender*’, ‘*Hemoglobin*’, ‘*Diabetes*’, ‘*Vascular*’, ‘*Heart disease*’, ‘*Hypertension*’, ‘*Obesity*’ ,‘*Cholesterol*’, ‘*Creatinine* (*Serum*/*Baseline*)’, ‘*eGFR*’, ‘*BP* (*sBP*/*dBP*)’, ‘*Smoking*’, ‘*BMI*’, ‘*Alcohol habits*’, ‘*Chewing habits*’, and ‘*Cancer Classification*’. The risk variables are handled by limiting the lab values, for example ‘*eGRF*’ is included only if the lab value is within the hospitalization range and ‘*vital_status*’ is ‘*censored*’, etc.

The data leaks were handled with the use of low-risk techniques such as handling the outliers and replacing the categorical data with either 0 or 1 like ‘gender’, ‘normal/abnormal’, or ‘cancer/nocancer’, which are crucial in the classification and prediction of renal cell carcinoma. Handling low-risk techniques includes ‘dropping identifier columns’, ‘dropping low-variance columns’ and ‘dropping highly correlated columns’ as they have no significance for predicting the results.

Other preprocessing steps include the feature selection technique which is used for handling the data leaks by selecting the features purely required for the training data. The feature correlation and important features were evaluated on the train–test split data to avoid the data leaks instead of being applied on the preprocessed dataset. Apparently the results of both the TCGA and Abu Dhabi dataset were restricted to not randomly achieve 99% or 100% accuracy and eventually other evaluation parameters will help to achieve the correct result even though the data is imbalanced.

### 4.3. Feature Engineering

Feature selection and classification are classified as feature engineering, which includes the selection of raw features and the creation of useful information for the same feature to increase the model accuracy. Feature engineering techniques use one or more features that eventually generate useful features for enhancing the prediction results. The feature engineering techniques include feature scaling and normalization, categorical encoding, binary clinical risk flag, interaction feature generation, feature selection, correlation pruning. Feature scaling and normalization scale the categorical features and normalize the features after scaling to the same scale as the dataset. Categorical encoding converts categorical data into the numerical format. The binary clinical risk flag in the healthcare domain classifies the patient risk status of high, medium, and low to binary numerical classification of two values and replaces them. Interaction feature generation calculates the value for a new feature using the combination of two or more features, such as calculating the BMI using the features of age, weight, and height of the patient. Feature selection in feature engineering includes the combination of one or more features for the finalization of the features. Correlation pruning removes the most correlated features of the dataset with all patients and keeps only minimal features for prediction so that it does not create any burden for the model.

Feature engineering refines the features by eliminating or generating vital features for prediction. This involves transforming raw clinical, imaging, and textual data into meaningful representations that enhance model performance [17]. For the clinical dataset, feature engineering includes the encoding of categorical data [18], a binary risk calculator, and the generation of interactive features. For image data, various feature engineering algorithms include deep feature extraction techniques, recursive feature elimination, and correlation pruning of the inter-correlated data among various features. Feature engineering is used to reduce overfitting and eliminate correlated features, missing values, etc. The highly correlated columns are those that have the maximum correlated values, such as creatinine_x_age’, ‘dm_yes’, and ‘cad_yes’. The refinement of the dataset facilitates the improvement of clinical features to boost the prediction results. The identifier column was eliminated for the model implementation, such as ‘patient_id’.

Feature scaling and normalization scaling adjusts the range of feature values so no other feature overpowers another. The normalization adjusts the range of data in the specified input range. For example, the feature scaling on ‘age’ uses the min–max scaling and categorizes them in different ranges. Whereas in the case of normalization, it is applied primarily on numerical data or encoding is required for categorical data. The min–max normalization scales the value in the range of [0–1] for features like ‘age’, ‘eGFRBaseline’, etc. Feature scaling and normalization is represented as follows, where $x_{scaling}$ is a scaling value and $x_{min}$, $x_{max}$ are the min and max values respectively:

(4) $$x_{scaling}\;=\;\frac{\left(x\;-\;x_{min}\right)}{\left(x_{max}\;-\;x_{min}\right)}$$

Categorical encoding converts the categorical data into numerical data based on the features; the encoding type is decided like binary for 0/1 encoding, nominal for one hot encoding, etc. For example, the feature ‘rbc’ with categorical values like ‘normal’ and ‘abnormal’ are converted to ‘0’ for normal and ‘1’ for abnormal.

Binary clinical risk flags are labeled as 0 or 1 as per the risk. For example, a feature ‘vital_status’ which has two values like ‘censored’ and ‘dead’ will be clinically represented as 0 for censored and 1 for dead, as the severity is very critical.

Interactive feature generation combines multiple features to draw a conclusion based on the results obtained. For example, the features like ‘last_preop_egfr’ and ‘first_postop_egfr’ calculate the patient’s condition after surgery and decide the post operative stay at hospital. Whereas the features like ‘age’, ‘gender’, ‘height’ and ‘weight’ are used to calculate the BMI of a patient which is an important feature in the clinical analysis of patients.

Feature selection, in feature engineering, identifies the features in model construction using the data from multiple features. The features were selected individually for all the feature sets and a filter is applied to identify the selected feature.

Correlation pruning removes the redundant features from feature sets. For example, features like ‘patient_id’ which are not required during the prediction process, or ‘vital_status’ which is an important feature for prediction, if considered, may result in data leaks.

### 4.4. Training of Dataset

The dataset for the classification of cancer or CKD and non-cancer or notCKD patients as CKD, which eventually leads to RCC prediction, was trained on 70% of the data and tested on 30% of the data. The Stratified Sampling technique is used to divide the sample sizes of CKD and notCKD patients with a specific percentage for the prediction of RCC.

The following method is used for Stratified Sampling technique:

*Stratified Random Sampling* = (*Sample Size*/*Population Size*) × *Population Groups* (5)

Here, the population groups are divided into CKD and notCKD whereas the sample size represents the number of CKD or notCKD patients and the population size is the total population. To avoid overfitting the dataset, the mean values are replaced with incomplete or null value columns, especially in the training dataset.

### 4.5. Machine Learning Classifiers

The machine learning classifiers identify the model performance based on the refined trained dataset [19]. The model was validated based on the prediction outcomes and types of machine learning classifiers. The machine learning classifier includes supervised learning methods such as Logistic Regression, Naïve Bayes, Decision Tree, Random Forest, Gradient Boost, XGBoost [20], and Multilevel Perceptron. Logistic Regression is used to map binary classification values or mapping input to the probability. Naïve Bayes classifies model probabilities based on the Bayes theorem. The Decision Tree uses model decisions based on multiple trees with multiple binary feature predictions. Random Forest is an ensemble approach [21] that uses multiple Decision Trees for higher accuracy. Gradient Boost also uses the ensemble learning methodology, which combines multiple Decision Trees and follows sequential learning. XGBoost is also called as second-order Gradient Boost algorithm used for highly efficient implementation [22]. The MLP is a deep learning algorithm with a feed-forward AI network. A comparison of these classifier models will help us validate the model that works well with the dataset.

The machine learning classifiers include various algorithms, namely, Logistic Regression, Naïve Bayes, Decision Tree, Random Forest, Gradient Boost, XGBoost, Multilevel Perceptron [20]. Logistic Regression primarily focuses on binary classification to categorize cancer and non-cancer predictions. The sigmoid function helps map the input features with a probability range of 0 to 1. The function can be represented as follows:

(6) $$\sigma \left(a\right)=\;\frac{1}{1\;+\;e^{-a}}$$

Naïve Bayes uses Bayes theorem with the conditional and independent variable features of the dataset. These textual features classify and label the probabilities. The Gaussian method is best suited for clinical datasets. Decision Tree relies on the partitioning of data into subsets by checking the features and their thresholds. The tree structure has a root node and decision nodes, similar to a leaf node. To determine the best feature splits, criteria such as the Gini Index [23] or information gain are employed. Decision Trees are rule-based predictions and thresholds are used for the markers and tumor dimension.

The Gini Index is represented as follows:

(7) $$GiniT=1-\sum_{i\;to\;m}\;P_{i}^{2}$$

where T is the i class and summation is computed over m number of classes.

Random Forest combines various Decision Trees using bagging techniques, and the final predictions can be evaluated using majority voting [24]. The clinical dataset performed well with the Random Forest algorithm to reduce overfitting. Feature importance helps enhance the predictability of the data. The Gradient Boost algorithm minimizes the error using an iterative function. The boosting algorithm uses a loss function to predict the result. XGBoost will use the upgraded Gradient Boosting methodology, while incorporating the techniques to reduce the overfitting and efficient training of models. Feature scaling and data handling helped improve the prediction results. XGBoost is integrated with an Explainable AI model.

(8) $$Output\left(x\right)=f_{n}\left(f_{n-1}\left(\ldots f_{1}\left(x\right)\ldots \right)\right)$$

where fi (xi) = σ (Wixi + bi).

Multilevel Perceptron (MLP) uses the feed-forward AI network with ReLU function between the input feature and output data. The MLP model is a combined model of feature selection and feature tuning to enhance predictive performance. The equation has a layer function f(x) with weight and constant values. The SHAP is applied on the best-fit model, which is Multilevel Perceptron here as per the comparison results of all the models.

### 4.6. Evaluation Parameters

Since the data is imbalanced in the TCGA and Abu Dhabi datasets, accuracy is not the primary evaluation metric for reviewing the best-fit model with the proposed methodology. Other parameters such as precision, recall, specificity, F1 score and MCC are reviewed which leads to a more accurate deduction of results. By this, the data imbalancing is handled with the metric-level technique. The primary evaluation metrics include combination of precision, F1 score and MCC for comparison with all the models across three datasets. The evaluation matrix includes accuracy, precision, recall/sensitivity, specificity and F1 score, as well as the Matthews Correlation Coefficient (MCC) [25]. Accuracy is the proportionate distribution of all positive and negative classifications. Precision is the classification of all positives of the prediction, giving the actual positive by the sum of the true positive and false positive (FP). Recall is the true positive (TP) rate proportionate to the actual positive and the sum of the true positive and false negative (FN) [14]. The F1 score is the mean of precision and recall. Matthews Correlation Coefficient (MCC) uses all four confusion metric components like TP, FP, TN, and FN [26] for the imbalanced dataset to give a balance evaluation:

(9) $$MCC=\frac{\left(TP\;\times \;TN\right)\;-\;(FP\;\times \;FN)}{\sqrt{\left(TP\;+\;FP)(TP\;+\;FN)(TN\;+\;FP)(TN\;+\;FN\right)}}$$

The MCC evaluates results when the data are highly skewed and imbalanced for calculation. The value ranges from +1 to −1, where +1 indicates perfect prediction, 0 corresponds approximately to random prediction, and −1 indicates complete disagreement.

The evaluation matrix such as accuracy which imprints how much a model is accurately predicting the result of if cancer is present or not. For medical diagnosis, identifying the false negative is critical, so recall is also an important consideration. Whereas the MCC is crucial for an imbalance dataset as three different datasets are considered.

The Explainable AI (XAI) is used to provide a clear understanding of the decisions made by the prediction model. XAI provides a view of black-box processing for prediction. The features used to boost the results are visualized using a diagrammatic representation. The XAI provides an overall review of the features used, the most impactful features in the prediction of disease, and the contribution of features in the prediction. The SHapley Additive exPlanation (SHAP) [27] is one of the most powerful algorithms used as an XAI to represent the model performance.

## 5. Results and Discussion

In the proposed research, the predictive model predicts renal cancer using clinical data from three different datasets. Each dataset was examined using machine learning classifiers, and the results were compared. The use of multiple datasets for renal cancer helps validate the prediction model. The proposed approach encompasses precise prediction, which helps enhance the cost-effectiveness and prediction results.

### 5.1. Results of the TCGA Dataset

The Cancer Genome Atlas (TCGA) [16] program offers a dataset for renal cancer prediction clinical data for research. The output after feature engineering on the TCGA dataset with the various machine learning classifiers is represented in the Table 3 below.

**Figure 3:**
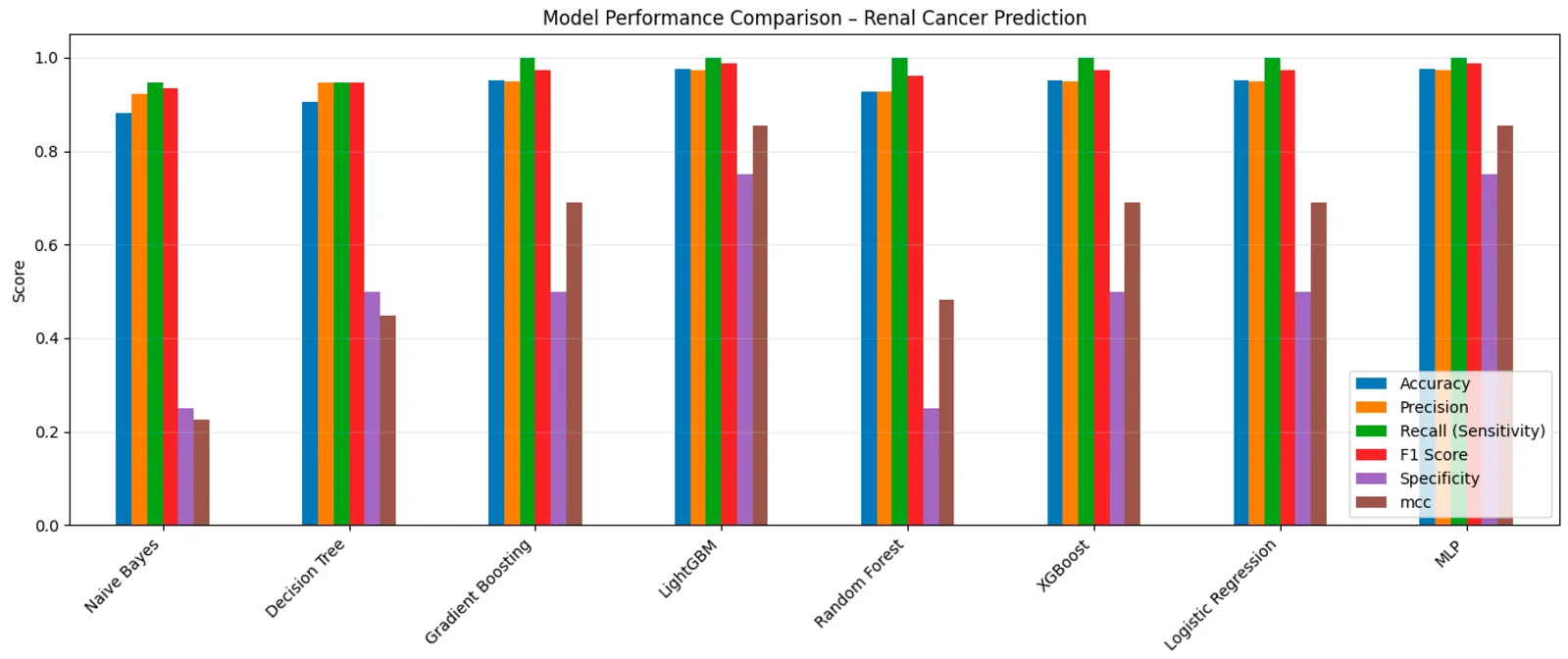
Model performance of all the algorithms using TCGA dataset.

The comparison in the above Table 3 shows the best performance of the multilevel perceptron model, with an accuracy of 98%, precision of 0.97%, recall of 100%, specificity of 75.5%, F1 score of 98%, and MCC of 0.85. The MLP employs feature engineering with clinical data to maximize its ability to predict renal cancer. The comparative analysis of the TCGA dataset-based model performance can be represented graphically in Figure 3 below.

**Figure 4:**
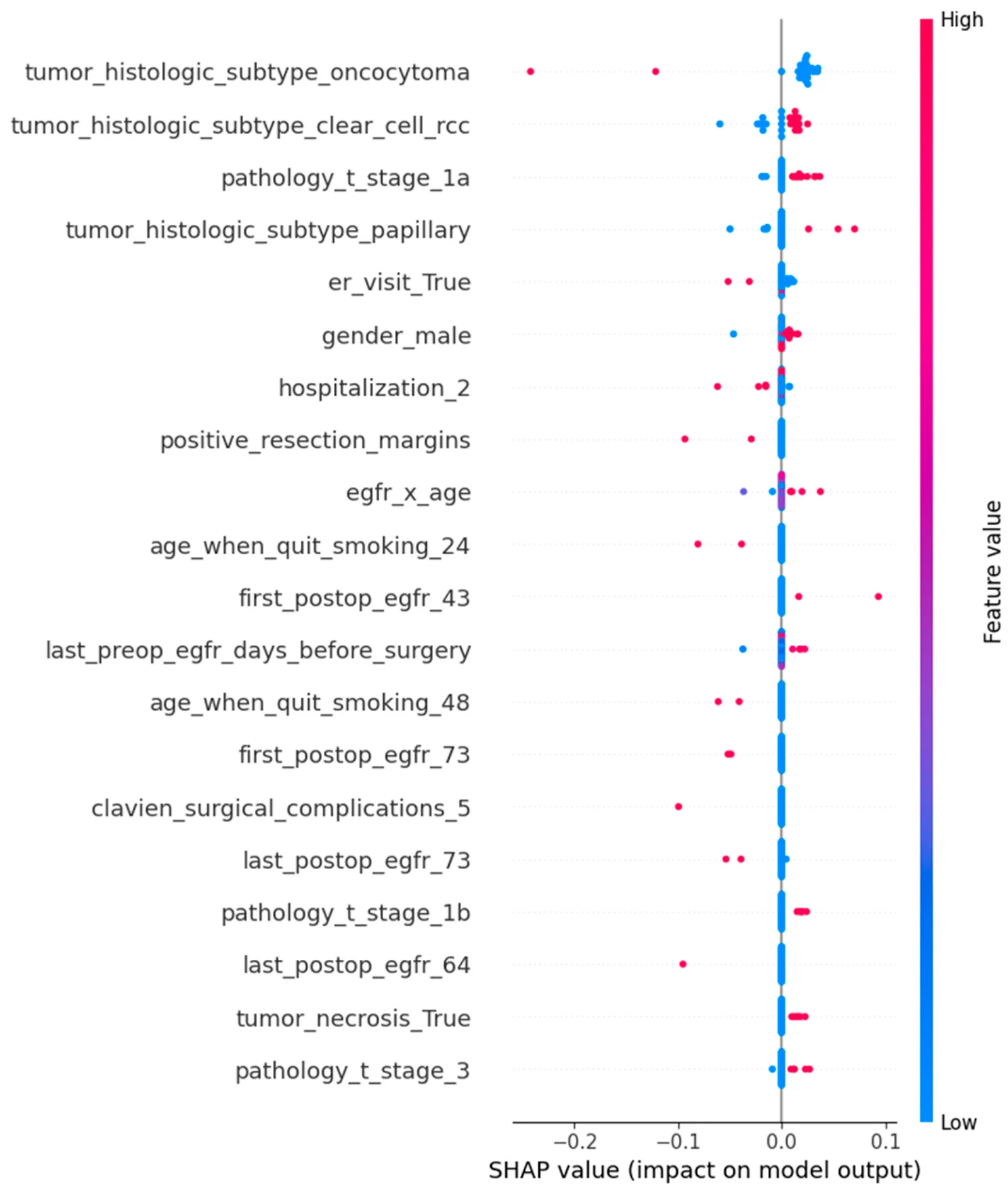
SHAP feature importance for the TCGA dataset.

The SHapley Additive exPlanation (SHAP) [28] method was applied on the TCGA dataset to determine the significance of features in predicting renal cancer. Figure 4 shows the feature distribution and its importance in renal cancer prediction.

Features such as the tumor histologic subtype for renal cell carcinoma and oncocytoma prominently impact the prediction of renal cancer. However, the pathology T stage 1a value and number of visits, along with the male sex, affected the overall results. In addition, features such as pathology T stage 3 and 1b less affect the prediction results. The SHAP values show features that have an impact on the prediction results.

### 5.2. Results of the Tawam Hospital in Al-Ain City (Abu Dhabi) Dataset

The Abu Dhabi dataset offers renal cancer patient information; the results after processing are shown in Table 4.

**Figure 5:**
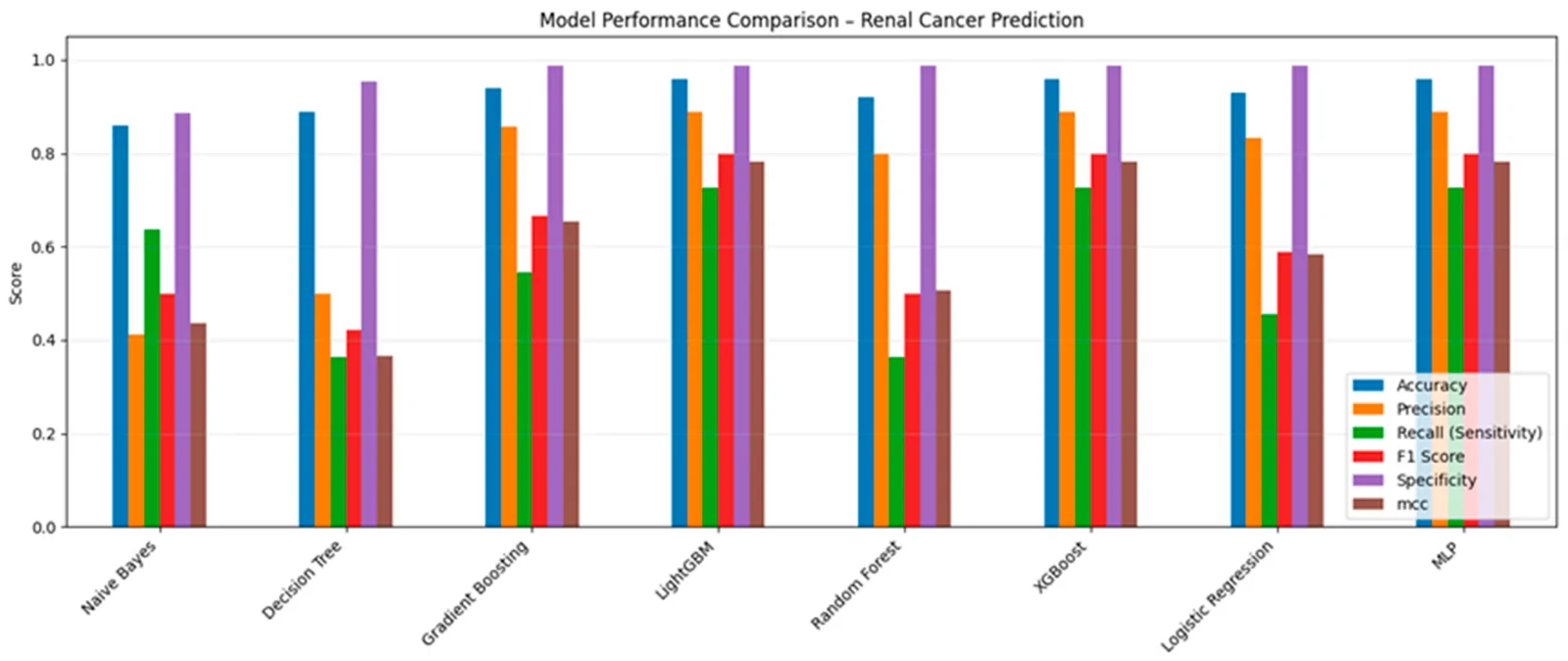
Model performance of all algorithms using Tawam Hospital in Al-Ain City (Abu Dhabi) dataset.

The comparison in the above table shows the best performance of the Multilevel Perceptron with 96% accuracy, precision 88%, recall 72%, specificity 98%, F1 score 80% and MCC 0.78, which is acceptable and shows the best performance compared to the other machine learning classifiers and is similar to the XGBoost model. MLP and XGBoost produced identical outcomes when applied to the feature-engineered clinical data used to predict renal cancer. The comparative analysis of the Tawam Hospital in Al-Ain City (Abu Dhabi) dataset-based model performance can be represented graphically in Figure 5 as below.

**Figure 6:**
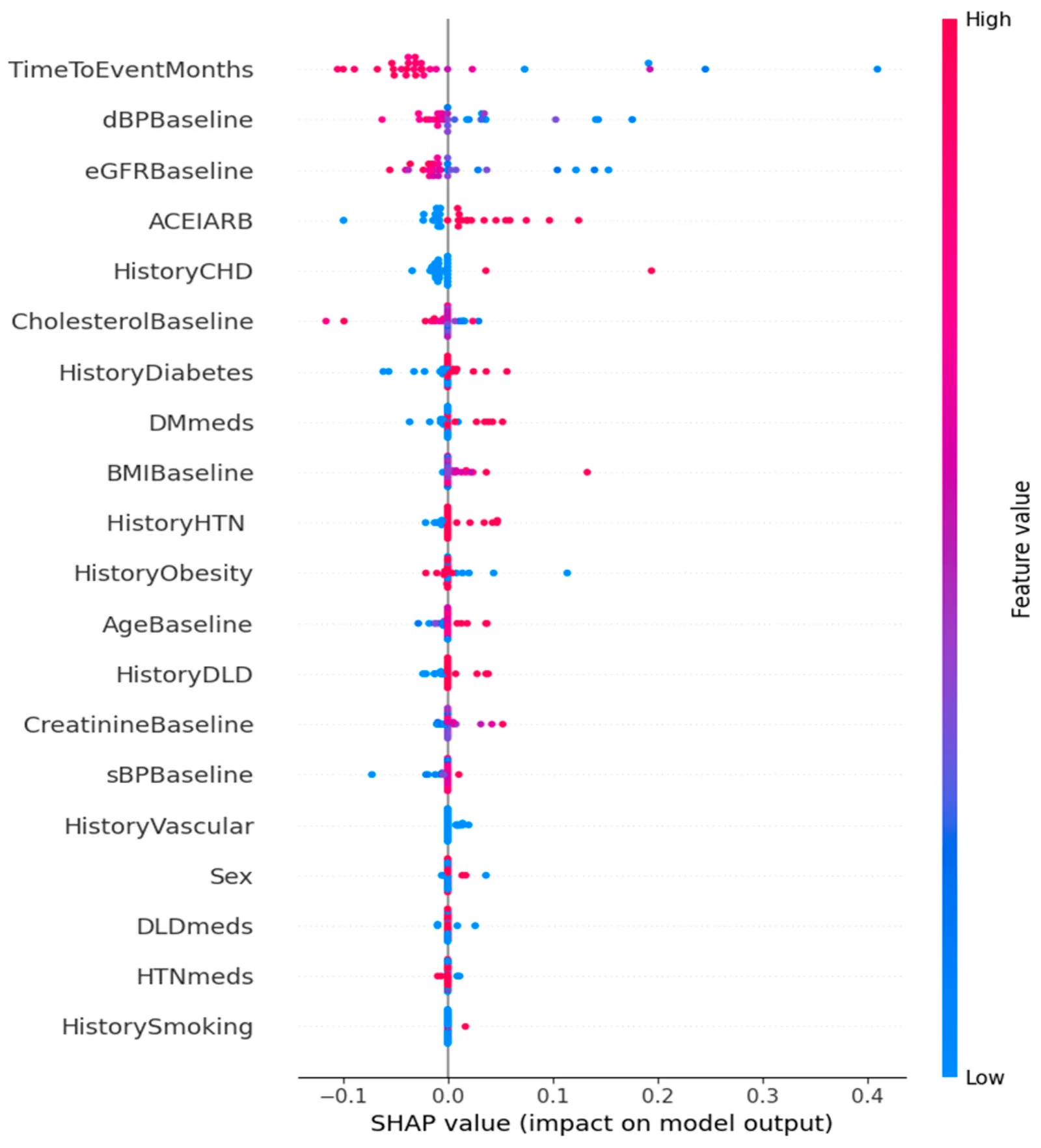
SHAP feature importance for the Tawam Hospital in Al Ain City (Abu Dhabi) dataset.

The SHAP method was applied to the Tawam Hospital in Al-Ain City (Abu Dhabi) dataset to determine significant features in predicting renal cancer. Figure 6 shows the feature distribution and its importance in renal cancer prediction.

SHAP shows important features that contribute to the prediction result of the hospital data, such as TimeToEventMonths, dbBaseline, and eGFRBaseline values. HistorySmoking, Sex, DLDmeds, and HTNmeds likely impacted the final prediction results.

### 5.3. Results of the India Dataset

The comparison in Table 5 shows the best performance of the MLP with predominant accuracy, precision, recall, specificity, and F1 score with an MCC of 1.00; MLP, Logistic Regression, Random Forest, Gradient Boost, and XGBoost yielded comparable outcomes for the Indian clinical dataset while performing renal cancer predictions.

**Figure 7:**
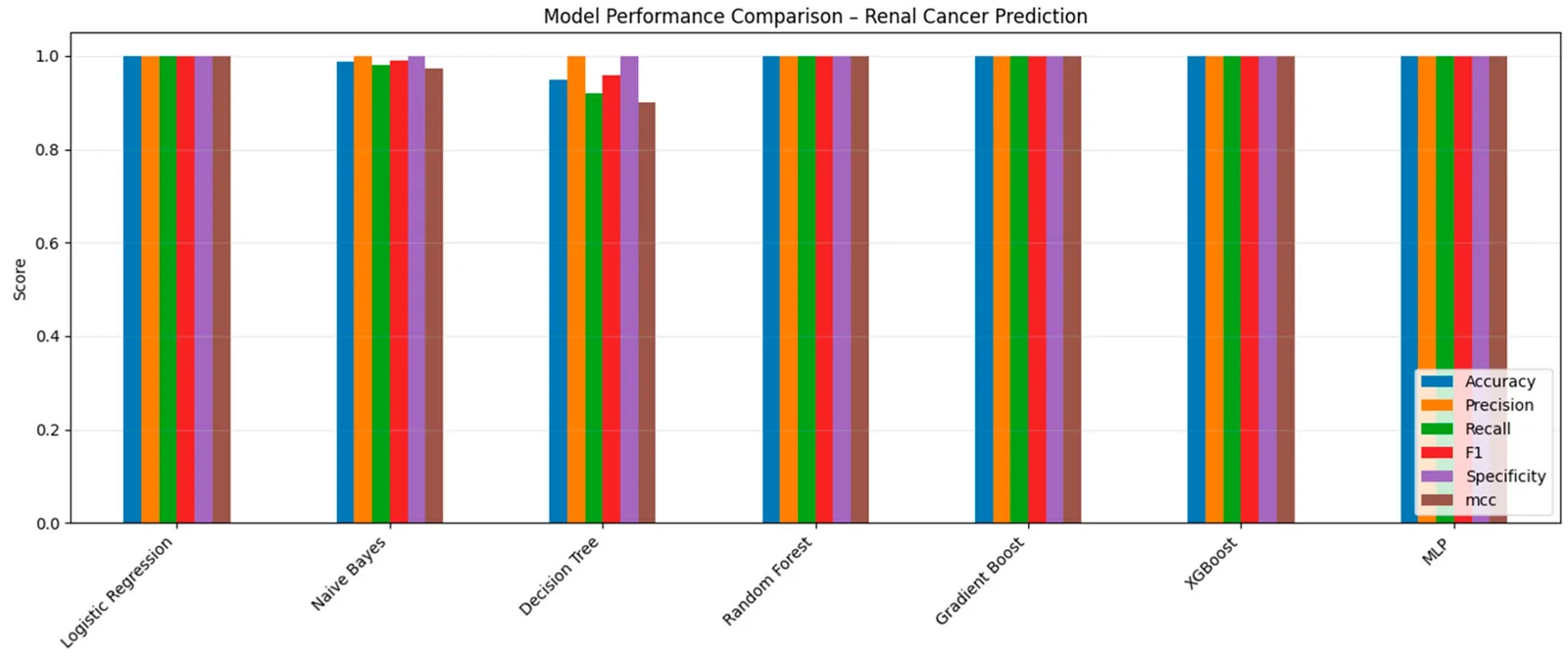
Model performance of all algorithms using the Indian dataset.

The performance of the India dataset exhibits a uniform performance because of few dataset constraints like the dataset size is small, the dataset is highly predictable due to deterministic combination of features, the data lacks noise, etc. The comparative analysis of the India dataset-based model performance can be represented graphically in Figure 7 as below.

**Figure 8:**
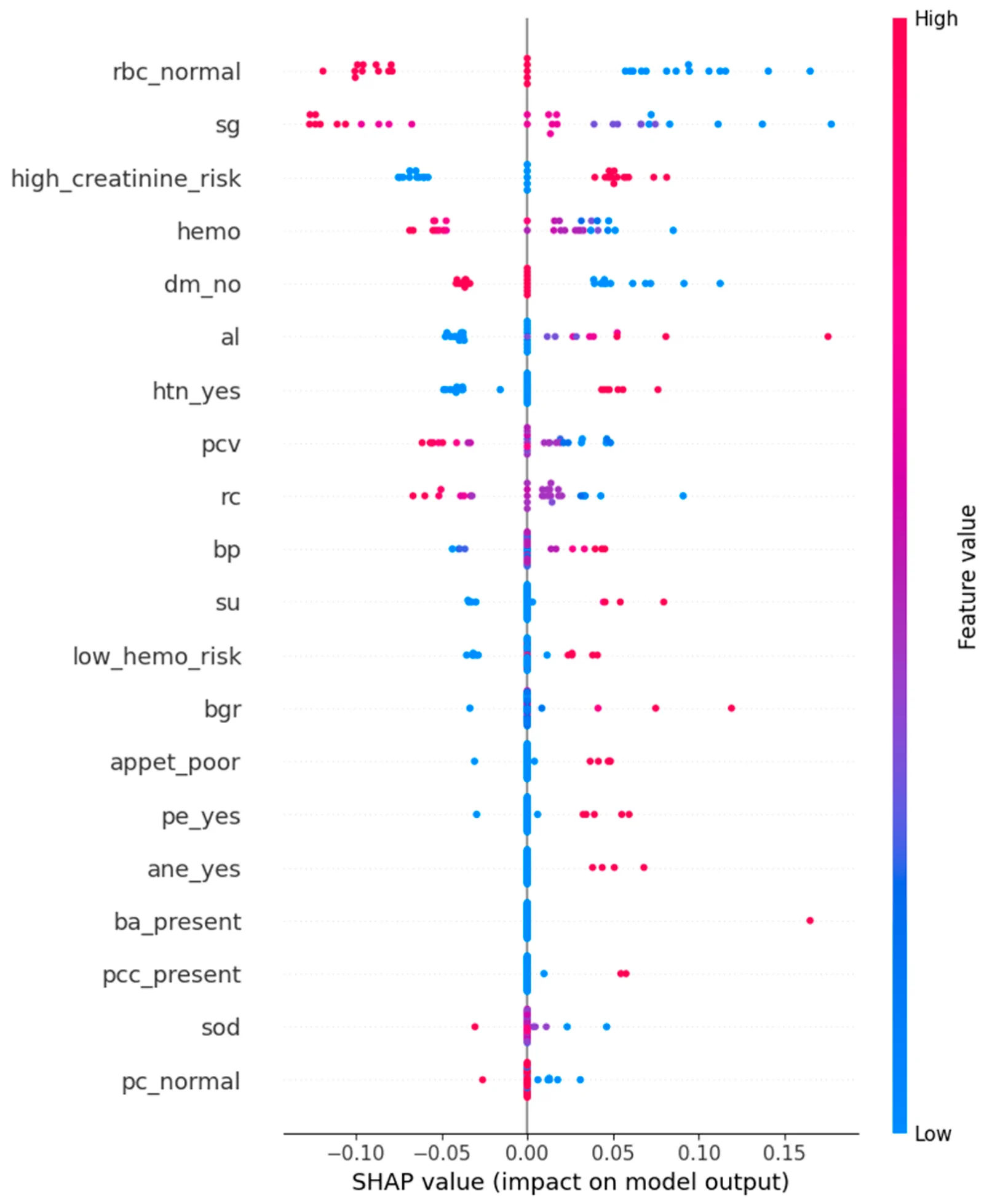
SHAP feature importance for the India dataset.

The SHAP method was applied to the Indian dataset to determine the significance of features in predicting renal cancer. Figure 8 shows the feature distribution and its importance in renal cancer prediction.

The SHAP figure above shows the impact of the rbc_normal and high_creatinine_risk values along with specific gravity, that is, sg, for the prediction of cancer. The feature values, such as papillary renal cell carcinoma that is pcc_present along with superoxide dismutase that is sod along with peritumoral pseudocapsule that is pc_normal, are less impactful for prediction.

The overall comparison of all the datasets can be represented as below in Table 6. along with the literature survey.

## 6. Conclusions and Future Work

Identifying renal cancer at an early stage within the healthcare sector opens up opportunities for researchers to apply AI to analyze medical data and enhance prediction outcomes. The results of this proposed research aim to forecast chronic renal cancer. The effectiveness of the outcomes was validated by evaluating the results across three distinct datasets using machine learning techniques. The integration of a Multilevel Perceptron and feature-engineered dataset proves an effective solution for all the renal cancer clinical datasets. The proposed model shows efficient results for the accurate prediction of individuals and subsequently paves the way for healthcare enhancement and disease prediction.

The model incorporates feature engineering methodologies, such as feature classification and feature selection, to select the appropriate set of features for the prediction of renal cancer. Feature engineering methodologies were used to encode the categorical data, generate binary risk flags, and create interactive features, which eventually helped boost the results to efficiently predict renal cancer. Correlation pruning eliminates the most correlated features from one or more feature sets, whereas outlier handling identifies and handles outliers.

The model demonstrated remarkable performance using feature engineering and machine learning classifiers. The evaluation consisted of a comparative analysis of three datasets for renal cancer prediction. The proposed model performs the best for the Multilevel Perceptron, which uses a feed-forward model for the prediction of renal cancer. Through the comparison of all three datasets, the results not only show the impressive performance of the MLP but also its effectiveness in the field of healthcare. Employing XAI aids in illustrating the significant features derived from the dataset for predicting renal cancer. It not only provides a visual representation of these features but also enhances our understanding of their importance in renal cancer prediction.

The chronic renal cancer model is cost-effective and efficient for prediction. The model can be checked for other chronic cancers. The use of multimodality along with clinical data boosts the prediction results and metastasis. The research areas can be explored not only in the healthcare domain but also in the research of other chronic cancers. The study is limited to a clinical dataset, whereas we can add multimodality to enhance the prediction results. Another limitation is on the use of a public dataset which has missing information, as the dataset is built from the demographics and lifestyle of people. Future work could consider using a dataset with the same demographics and comparing the results to improve the relatability of the proposed model.

## Figures and Tables

**Table 1:** Review of the literature.

Reference Number	Dataset	Feature Selection/Extraction Methods	Classification/Methodology	Performance Evaluation	Inferences
[11]	UCI Machine Learning Repository [12], including the “Iris”, “Hepatitis”, “Breast Cancer Wisconsin (Diagnostic)” and “Spambase”	Feature selection—MLP Ensemble Voting Classifier—RF + SVM	MLP feature selected Iris—1 feature Hepatitis—15 Breast Cancer—15 Spambase—36	Accuracy: Iris—1.00 Hepatitis—0.90 Breast cancer—1.00 Spambase—0.95	The dataset has optimal values for prediction, which is why it provides predominant results.
[8]	Tawam Hospital in Al-Ain City (Abu Dhabi, United Arab Emirates) [13] Kidney Disease India Dataset [12]	Feature engineering (Hybrid SVM + RFE)	SVM, DT, RF, K-NN, GNB, LR, and MLP, the hybrid BO–XGBoost model	Abu Dhabi dataset (Accuracy—0.96, Precision—0.83, Recall—0.62, F1 score—0.71) and India dataset (1 for all)	The Tawam dataset provided optimal results for renal cancer, and the Indian dataset provided predominant results.
[14]	Hospital of Qingdao University	Preprocessing—Gaussian Filter	LR, SVM, RF, XGBoost, LightGBM, Gaussian NB, KNN	Logistic Regression: AUC = 0.8154 and accuracy = 0.787	The comparative results are very limited.
[15]	Korean Renal Cell Carcinoma Database (KORCC)	Data Augmentation—SMOTE Feature selection—ElasticNet	Logistic, SV, DT, RF, Naïve, XGBoost, NGBoost, LightGBM, CatBoost	Recurrence and Survival of patient with high accuracy of 0.94 and F1 score of 0.97	Data imbalance problem due to follow-up cases for 10 years. Developed model only included Korean population.
[2]	UCI Machine Learning Repository [12] for Chronic Renal Cancer	Filters, wrappers, and embedding approaches	ANN, RF, DT, SVM, Naïve Bayes, LR	The highest score is achieved by ANN, that is, 100%, whereas LR and SVM achieved 96% and 82%, respectively	The suggested model has a perfect passing rate. The dataset provides predominant results.
[3]	UCI Machine Learning Repository [12] for Chronic Renal Cancer	Biology-based optimization BBO algorithm and XGBoost classifier	Decision Tree, SVM, Multilevel Perceptron, KNN, CNN, Random Forest, Logistic Regression, XGBoost	XGBoost result Accuracy—99.16%, Precision—100%, Recall—98.68% and F1 score—99.33%	The XGBoost classifier, along with BBO, yielded optimal results.
[4]	The chronic kidney disease dataset	k-PCA	PCA, MLP, Genetic Algorithm	Accuracy: Breast cancer—99.12% Parkinson’s disease—94.87% and Chronic kidney disease—100%	PCA for renal cancer proved to be predominant.
[7]	UCI Machine Learning Repository [12] for Chronic Renal Cancer and Hungarian datasets	Proposed EBO-IANN algorithm	NN, MKSVM, EANFIS algorithms	Precision of 98.9% and 95.9% for the CKD and Hungarian datasets	The existing techniques produce lower f-measure for both datasets.

**Table 2:** Dataset comparison table.

Dataset	TCGA		Abu Dhabi		India	
**Number of Subjects**	210		491		400	
**Number of Features Before Preprocessing**	60		22		25	
**Number of Features After Preprocessing**	32		22		26	
**Class Distribution**	Cancer	NoCancer	Cancer	NoCancer	Cancer	NoCancer
	192	18	56	435	250	150
**Missing Value Percentage**	0%		0%		0%	
**Number of Selected Features**	32		22		25	
**Training Sample Sizes**	Cancer	NoCancer	Cancer	NoCancer	Cancer	NoCancer
	134	13	39	305	175	105
**Testing Sample Sizes**	Cancer	NoCancer	Cancer	NoCancer	Cancer	NoCancer
	58	5	17	130	75	45
**Data Source**	TCGA		Tawam Hospital in Al-Ain City (Abu Dhabi)		India	
**Prediction Target**	ccRCC		CKD		CKD	

**Table 3:** Comparative results of all classifiers using TCGA dataset.

	Accuracy	Precision	Recall	Specificity	F1 Score	MCC
Logistic Regression	0.95	0.95	1.00	0.50	0.97	0.85
Naïve Bayes	0.88	0.92	0.94	0.25	0.93	0.22
Decision Tree	0.90	0.94	0.94	0.50	0.94	0.44
Random Forest	0.92	0.92	1.00	0.25	0.96	0.48
Gradient Boost	0.95	0.95	1.00	0.50	0.97	0.68
XGBoost	0.95	0.95	1.00	0.50	0.97	0.53
Multilevel Perceptron	0.98	0.97	1.00	0.75	0.98	0.85

**Table 4:** Comparative results of all classifiers using Tawam Hospital in Al-Ain City (Abu Dhabi) dataset.

	Accuracy	Precision	Recall	Specificity	F1 Score	MCC
Logistic Regression	0.93	0.83	0.72	0.98	0.58	0.58
Naïve Bayes	0.86	0.41	0.63	0.88	0.50	0.40
Decision Tree	0.89	0.50	0.36	0.95	0.42	0.36
Random Forest	0.91	0.80	0.36	0.98	0.50	0.50
Gradient Boost	0.94	0.85	0.54	0.98	0.66	0.65
XGBoost	0.96	0.88	0.72	0.98	0.80	0.78
Multilevel Perceptron	0.96	0.88	0.72	0.98	0.80	0.78

**Table 5:** Comparative results of all classifiers using the Indian dataset.

	Accuracy	Precision	Recall	Specificity	F1 Score	MCC
Logistic Regression	1.00	1.00	1.00	1.00	1.00	1.00
Naïve Bayes	0.98	1.00	0.98	1.00	0.98	0.97
Decision Tree	0.95	1.00	0.92	1.00	0.95	0.92
Random Forest	1.00	1.00	1.00	1.00	1.00	1.00
Gradient Boost	1.00	1.00	1.00	1.00	1.00	1.00
XGBoost	1.00	1.00	1.00	1.00	1.00	1.00
Multilevel Perceptron	1.00	1.00	1.00	1.00	1.00	1.00

**Table 6:** Comparative results of all datasets along with literature review results.

Datasets	Literature Survey—TCGA	Proposed Model-TCGA	Literature Survey—Tawam Hospital in Al-Ain City (Abu Dhabi)	Proposed Model—Tawam Hospital in Al-Ain City (Abu Dhabi)	Literature Survey—Kidney Disease India	Proposed Model—Kidney Disease India
Results						
Accuracy	0.99	0.98	0.96	0.96	1.00	1.00
Precision	1.00	0.97	0.83	0.88	1.00	1.00
Recall	0.98	1.00	0.62	0.72	1.00	1.00
F1 score	0.99	0.98	0.71	0.80	1.00	1.00

## Data Availability

The datasets used to develop the clinical data prediction model were obtained from publicly available sources, including The Cancer Genome Atlas (TCGA) [16], the Tawam Hospital cancer dataset from Al-Ain City, Abu Dhabi [13], and the UCI India dataset [12]. These datasets contain relevant clinical and patient-related information for cancer research and were accessed from their respective online repositories. No new clinical data were generated during this study.
